# Length- and Usage-Weighted Indices for Representative Route Extraction from Trajectory Data

**DOI:** 10.3390/s26041114

**Published:** 2026-02-09

**Authors:** Choongheon Yang

**Affiliations:** Department of Highway and Transportation Research, Korea Institute of Civil Engineering and Building Technology, Goyang-si 10223, Republic of Korea; chyang@kict.re.kr

**Keywords:** vehicle trajectory data, representative path extraction, weighted metrics, sensor-based transportation networks, intelligent transportation system

## Abstract

**Highlights:**

**What are the main findings?**

- Three weighted indices (Lw, ALw, Ow) mitigate short-link bias and recalibrate overlap effects.
- Weighted indices reduce structural variance by 20–30% and enhance route grouping stability, and Spearman correlations (ρ = 0.863, 0.071, 0.189) confirm robust calibration without distortion.

**What is the implication of the main finding?**

- More reliable representative links/paths can improve VKT estimation, congestion diagnostics, and large-scale trajectory analytics in heterogeneous urban networks.
- The weighted framework is practical and reproducible, supporting integration into ITS and navigation services for data-driven planning and policy.

**Abstract:**

This paper introduces weighted indices—link passing ratio adjusted by length, average link usage ratio weighted by frequency and length, and path overlap weighted by length and usage—to improve representative path extraction from large-scale vehicle trajectory data. Conventional indices often overstate the representativeness of short links, leading to biased path similarity and unstable grouping. The proposed indices explicitly down-weight short segments such that routes with many small links no longer appear falsely similar. Using data from 18,205 real-world urban trajectories, the weighted indices reduced short-link bias by 20–30% and increased the stability of representative path grouping by 15–30% compared with conventional metrics. Distribution of comparisons confirmed that the weighted indices consistently capture the structural characteristics of real-world GPS-based trajectories, reflecting stable link usage and overlap patterns. These improvements were evaluated on a refined subset comprising 12,540 link-level observations and 8320 route pair comparisons, ensuring statistical robustness and consistency. These improvements are expected to enhance downstream applications such as estimations of vehicle kilometers traveled, congestion diagnostics, and sensor-based mobility services. The findings demonstrate that refining trajectory similarity metrics at the link level has direct implications for intelligent transportation systems, supporting accurate analysis and practical decision-making in large-scale urban mobility management.

## 1. Introduction

Recent transportation policy frameworks increasingly emphasize data-driven mobility analysis as a foundation for sustainable and integrated transportation planning, highlighting the practical relevance of representative route extraction. The rapid expansion of trajectory data collected through commercial navigation systems has reshaped the field of transportation analysis. Such data provide detailed insights into travel behavior, route choice, and network performance, thus enabling congestion monitoring, estimation of vehicle kilometers traveled (VKT), and emission modeling. However, handling large-scale raw trajectories poses challenges for computational efficiency and representativeness, requiring methods that can extract a manageable set of representative routes that still preserve the essential travel patterns.

A long-standing issue in representative route analysis concerns managing route similarity. Conventional logit-based models, such as the path size logit (PSL), were designed to correct probability distortions due to route similarity. Duncan et al. [1] propose the adaptive PSL (APSL) model, which maintains internal consistency between route probabilities and path size terms in large-scale global positioning system (GPS) datasets. Concurrently, researchers have focused on critical infrastructure identification and trajectory-based clustering. Wang et al. [2] introduce a framework for discovering the most representative routes from large-scale trajectories, addressing the need for computationally efficient algorithms in big-data contexts. These studies highlight both the methodological and computational demands of representative path selection.

The rise of machine learning has broadened methodological options. Sroczyński et al. [3] show that traffic conditions can be predicted by machine learning models with accuracy commensurate with that of complex microscopic simulations, indicating the potential of data-driven approaches to complement or replace conventional traffic models. Complementarily, Hussain et al. [4] propose a novel framework for trajectory clustering without complete map matching, highlighting the role of preprocessing techniques in ensuring data quality and reliability for downstream analysis.

Despite these advances, existing methods still exhibit limitations. Short-link bias, inconsistent treatment of overlap, and insufficient weighting of structural network properties often distort representative route identification. To address these gaps, this paper proposes a new set of weighted indices, namely the weighted link passage ratio (Lw), weighted average link usage ratio (ALw), and weighted overlap ratio (Ow), that explicitly account for link length and usage frequency. With 18,205 real-world trajectories collected in Bucheon City, Republic of Korea, the proposed indices were empirically validated against conventional metrics, demonstrating improved robustness in representative route grouping and link selection.

The contributions of this study are twofold: (i) advancing the methodological basis for representative path extraction and (ii) offering practical insights for intelligent transportation systems, VKT estimation, and sustainable mobility planning.

## 2. Literature Review

### 2.1. Route Choice Modeling and the Path Overlap Problem

Trajectory data are central to both academic transportation modeling and real-world policy implementation. A fundamental challenge in route choice analysis is overlap among paths, which distorts choice probabilities when alternative paths share common segments. To address this issue, the PSL model was introduced, followed by extensions such as the generalized PSL and APSL, thereby aligning route choice probability and path size factors. Tan et al. [5] incorporate frequency variability into public transport route modeling, and Dixit et al. [6] compare multiple definitions of path overlap, including link-, segment-, and node-based measures, to identify criteria that best capture actual traveler behavior. Recently, Wang et al. [7] have applied ensemble machine learning models to surpass the scalability limitations of conventional logit-based methods. Despite these advances, PSL-based models remain difficult to scale in large networks or with high-volume trajectory data, highlighting the need for alternative, more efficient representations of route patterns. In parallel, the problem of route diversification under overlap constraints has been formalized in database and spatial computing research. Luo et al. [8] propose a diversified top-k route planning framework that explicitly controls similarity between candidate paths using length-weighted overlap thresholds, ensuring that selected routes are both representative and distinct.

### 2.2. Trajectory Clustering and Similarity Metrics

As trajectory datasets have grown in size and resolution, accurately measuring route similarity has become essential for representative path analysis. Conventional similarity measures, such as dynamic time warping, edit distance on real sequences, and longest common subsequence (LCSS), have been widely used for comparing spatiotemporal trajectories [9]. Dorosti et al. [10] introduce a spatiotemporal similarity metric that integrates geographic coordinates, timing, and sequence information, significantly improving both accuracy and scalability in GPS-based road networks. Similarly, Liu et al. [11] introduce a frequent-route identification method using link passing ratio (LPR) data, combining the Snake algorithm to mitigate data sparsity while uncovering recurrent route structures. These studies highlight the demand for similarity metrics that are robust under diverse sensing conditions and scalable to city-scale networks.

These methods typically prioritize sequence alignment or geometric proximity but often overlook the structural characteristics of the road network, such as link importance or directionality. To enhance interpretability and clustering accuracy, several researchers propose hybrid approaches. For example, Advani et al. [12] introduce a bilevel trajectory clustering model that accounts for path overlap and network constraints. Kim and Mahmassani [13] have combined LCSS with density-based clustering to analyze New York City trip patterns. More recently, deep learning-based models such as SimRN [14] and contrastive graph learning approaches [15,16] are used to improve similarity detection in complex networks. However, these approaches often depend strongly on high-frequency GPS traces and are sensitive to data sparsity or measurement noise, particularly in urban tunnels or at intersections. Additionally, they typically treat all matched links equally while ignoring their functional relevance in the traffic network. To address these limitations, the proposed Ow index integrates both the physical length and empirical usage frequency of overlapping links, providing a more conservative and functionally grounded measure of trajectory similarity. This novel formulation enables robust clustering even in sparse, heterogeneous datasets and improves the interpretability of representative route sets in real-world settings.

### 2.3. Link-Level Representation and Key Road Extraction

Although trajectory-level analysis contains rich behavioral insights, many practical applications, such as infrastructure monitoring, policy evaluation, and traffic simulation, require link-level representations of network usage. Accordingly, recent studies have focused on identifying key links that disproportionately influence traffic flow or network stability. For example, Feng et al. [17] have constructed a weighted complex network using GPS data to detect critical roads, and Zhang et al. [18] analyze how limited path overlap may enhance network resilience during disruptions. Yang and Moon [19] propose a representative link-based framework to estimate total VKT. Their results indicate that the reliability of representative links directly influences the accuracy of large-scale traffic performance indicators, reinforcing the importance of robust link-level metrics. These efforts underscore the practical need for reducing high-dimensional trajectory data to interpretable, scalable link-level indicators. However, existing metrics often overlook the varying importance of links based on their length, usage frequency, or function in linking origin–destination (OD) pairs.

As a result, minor local streets with high traversal counts can be mistakenly overrepresented as critical links. To overcome this issue, this paper proposes two weighted indicators, Lw and ALw, that incorporate both the physical length and relative usage of each link. These indices permit detection of structurally significant links that are both frequently used and physically substantial, thereby improving the reliability of representative link selection. This approach is particularly beneficial for applications such as VKT estimation, link-based congestion diagnostics, and AI-driven traffic monitoring systems.

### 2.4. Global Policy Frameworks and Trajectory Data Utilization

The increasing availability of trajectory data has led international institutions to integrate such data into strategic frameworks. The OECD/ITF [20,21] emphasizes the role of trajectory data in behavioral analysis and network performance monitoring while calling for standardization and privacy safeguards. The UN [22] highlight trajectory-based congestion monitoring as vital to meeting the Sustainable Development Goals (SDGs) and carbon-neutral transport goals. The EU Sustainable and Smart Mobility Strategy [23] promotes big data-driven intelligent transportation systems (ITSs) as a core pillar, a position further supported by JRC urban mobility studies [24]. In the U.S., the U.S. Department of Transportation ITS JPO [25] and National Highway Transportation Safety Administration [26] have demonstrated the benefits of real-time route data in improving road safety and traffic efficiency. In Asia, the Smart Mobility Challenge of Japan [27] and the ITS Master Plan of Korea [28,29] highlight the growing reliance on trajectory-based platforms for smart urban mobility.

### 2.5. Research Gap and Study Motivation

Although these developments represent substantial progress, several limitations remain within current trajectory-based indices:

1. Overweighting of short links.
2. Oversimplified route similarity assessments.
3. Nonuniform identification of representative links.

These structural issues impair both academic model accuracy and policy-level decision-making effectiveness.

To address this gap, this paper introduces a suite of weighted representativeness indices that collectively integrate link length, usage frequency, and structural similarity. These indices aim to extract more reliable and behaviorally plausible representative routes. The proposed framework advances both academic refinement and practical applicability, offering a robust foundation for high-resolution traffic analysis, network performance evaluation, and data-informed mobility policy.

## 3. Methodology and Development of Weighted Indices

### 3.1. Background of Indicator Development

In extracting representative routes from large-scale trajectory datasets, the priority is not merely computational simplicity but rather practical applicability and reliability in real-world contexts. Representative routes and links must accurately reflect the underlying functions of the traffic network, road characteristics, and observed travel patterns to support meaningful applications in traffic management, congestion diagnostics, and policy deployment. To this end, this paper proposes a novel set of weighted indices that explicitly incorporates two critical factors, link length and usage frequency, which together improve the consistency of route similarity evaluation. Moving beyond simple frequency-based aggregation, the proposed indices are designed to answer questions such as “Which links play a structurally significant role in actual traffic patterns?” and “To what extent do multiple routes form a consistent and interpretable networkwide structure?”

**Figure 1:**
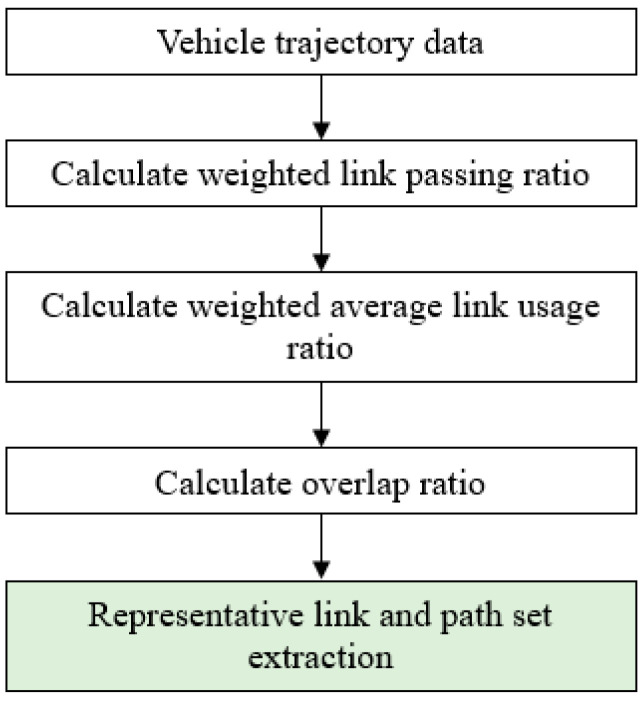
Workflow for computing weighted representativeness indices.

Figure 1 depicts the conceptual structure of the proposed weighted representativeness indices. The diagram illustrates how the proposed indices (Lw, ALw, and Ow) integrate key factors such as link length and usage frequency to support route similarity evaluation. Each indicator reflects a distinct aspect of route representativeness, and their combined application supports a robust evaluation of representative links and paths in sensor-based trajectory data.

#### 3.1.1. Data Processing

**Figure 2:**
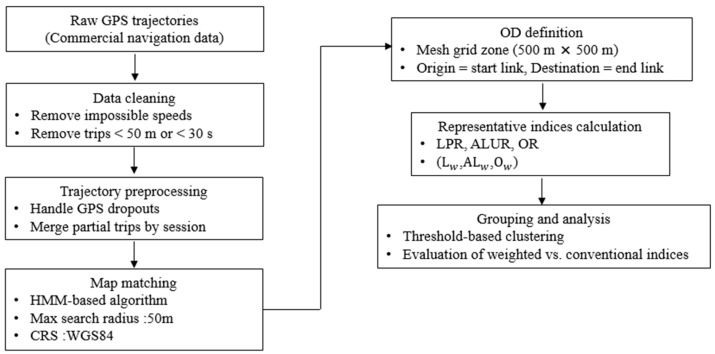
Workflow for data processing and representative path extraction using large-scale GPS trajectories.

In this study, only large-scale GPS trajectories obtained from commercial navigation systems were used. Although the nominal sampling rate of the devices is below 1 s, the actual recording intervals are irregular, with approximately 65% of points sampled within 1 s and approximately 95% within 1 min. Figure 2 depicts the overall data processing flow.

Representative preprocessing procedures commonly applied to large-scale commercial GPS trajectory data were employed. Trips shorter than 50 m or 30 s were excluded, and implausible speeds above 180 km/h were filtered out. Missing points were interpolated when involving fewer than two consecutive samples, whereas longer gaps resulted in trip segmentation. Map-matching followed a standard hidden Markov model (HMM) framework, applying commonly used default parameter configurations without manual tuning. Turn restrictions and one-way rules from the Korea Transport Database (KTDB) road database were applied to ensure realistic network topology. Duplicate and partial trajectories were merged using vehicle session identifiers. OD zones were defined by a 500 m × 500 m mesh grid, with the first and last matched links assigned as the origin and destination, respectively.

**Figure 3:**
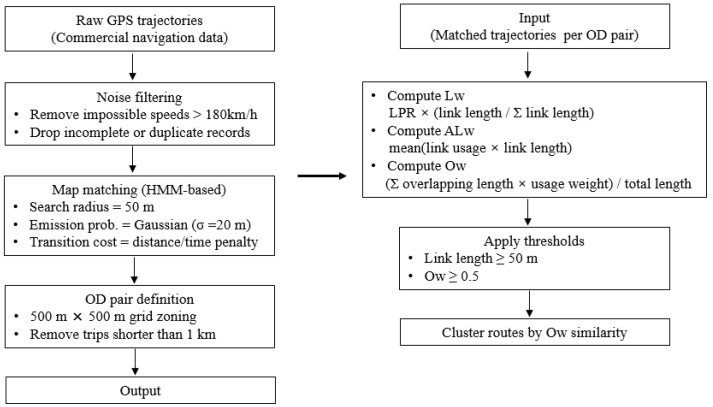
The end-to-end workflow for representative route extraction using weighted indices. The pipeline integrates HMM-based map-matching, weighted index computation, and threshold-based route grouping. Input–output data types and parameter settings correspond to those summarized in Table 1 and Table 2. The overall computational complexity is O (N log N + *k*^2^), where (N) denotes the number of matched trajectories and (*k*) is the number of candidate routes per OD pair.

According to the parameters summarized in Table 1, each stage of the processing pipeline was sequentially implemented to ensure reproducibility. Raw GPS data were filtered using plausibility rules (e.g., removing speeds exceeding 180 km/h and trips shorter than 50 m or 30 s). Missing points involving fewer than two consecutive samples were linearly interpolated, whereas longer gaps resulted in trajectory segmentation. Map-matching was performed using a standard HMM-based algorithm with default parameters, as described previously. The matched trajectories were converted into link sequences and grouped by OD pairs defined on a 500 m × 500 m grid. Routes with fewer than 20 observations were excluded to ensure statistical reliability, and sequences uniqueness was determined using a 10% length-difference threshold. Similarity scores were computed for all candidate routes, and representative paths were identified using a max-score selection strategy. The overall workflow is illustrated in Figure 3. Although the map-matching step utilizes a standard HMM-based approach with default parameters, potential matching uncertainty may influence downstream indices (e.g., link usage or overlap ratios); this effect was not quantitatively assessed here and will be addressed in future work. Because all representativeness indices are computed on map-matched link sequences rather than raw GPS coordinates, the impact of point-level positioning errors is substantially attenuated. In addition, the proposed length-weighted formulations further suppress potential distortions arising from short or fragmented links that are more sensitive to map-matching uncertainty.
**Algorithm 1.** Weighted representative route extraction frameworkInput:  T = set of all trajectories from commercial navigation sensors  N = road network with link attributes (ex: length, direction, type)  OD = set of valid origin-destination pairs Output:  R = set of representative routes with weighted indices Procedure: 1. Preprocessing trajectoriesRemove trips with length < 50 m or duration < 30 sFilter outliers (speed < 180 km/h)Interpolate GPS gaps ≤ 2 points2. Perform map-matching using HMMSearch radius = 50 mApply one-way and turn restrictions from KTDB DB3. Construct OD pairsDefine 500 m × 500 m grid meshAssign first and last matched links as origin and destination4. Generate candidate route sets for each OD pairDistinct link sequences from map-matched trajectoriesExclude OD pairs with <20 valid trajectories5. Compute representative indicesFor each link *l* in route r: (LPR, Lw, ALUR, ALw)For each route pair (*r*1, *r*2): (Ow)6. Group similar routesThreshold τ_OR ∈ [0, 1]If Ow ≥ τ_OR→merge into one representative cluster7. Output R = {*r*1, *r*2, …, *r*k} (top-k representative route)Compute coverage and VKT estimation errorEnd. Computational complexity: Map-matching: O (|T| × log|N|) Index computation: O (|OD| × |L|) Clustering (by Ow): O (|OD| × *k*^2^), where *k* ≤ 15

**Table 1:** Parameter settings applied in data preprocessing, map-matching, and representative route extraction. All parameters correspond to those specified in Figure 3 and Algorithm 1.

Step	Parameter/Methodology	Value/Rule
Data source	GPS trajectories from navigation logs	1–5 s sampling interval
Coordinate system	World Geodetic System	WGS84
Map-matching algorithm	Hidden Markov model (HMM)	Max search radius = 50 m
Outlier removal	Speed threshold	>180 km/h removed
Minimum trip length	Length/duration cutoff	>50 m and >30 s retained
GPS dropout handling	Missing ≤2 points: linear interpolation; >3 points: trip split	Rule-based
Duplicate handling	Trip ID-based unique trip	Duplicate applied
OD zone definition	500 m × 500 m mesh grid	Based on national road network
Turn restrictions	Applied	From KTDB

**Table 2:** Supplement parameters used in the computation of weighted indices.

Parameter	Symbol	Description	Value/Rule	Note
Ow threshold	τ_OR	0.4–0.5	Tuned empirically	Tuned empirically
Minimum OD samples	Nmin	Required number of trajectories per OD pair	20	For statistical reliability
Top representative routes	k	Number of top-k representative routes extracted	5	Used in coverage evaluation
Weighting factor	ε	Minimum relative difference between routes	10% length difference	Used in candidate filtering
Grid matching tolerance	δ	50 m	For OD boundary adjustment	For OD boundary adjustment

#### 3.1.2. Trajectory Preprocessing

Because the sampling interval of commercial GPS trajectories is inherently irregular, missing points were not identified based on absolute sampling frequency. Instead, missing points were defined as abnormal temporal gaps relative to the local sampling pattern within each trajectory. Short gaps corresponding to two or fewer consecutive missing samples were linearly interpolated to preserve trajectory continuity, whereas longer gaps were treated as trajectory breaks and resulted in segmentation. This rule-based approach distinguishes natural irregular sampling from true data loss and prevents artificial inflation of link usage.

Beyond missing-point handling, raw GPS data were preprocessed to ensure validity and internal consistency prior to similarity computation and index extraction. Each trajectory was filtered to exclude trips shorter than 50 m or 30 s, and partial sessions were merged only when belonging to the same vehicle ID and exhibiting directional continuity. Although these preprocessing procedures are commonly adopted in large-scale trajectory mining, potential residual duplication or minor bias in link usage frequency may remain. Future work will incorporate dropout-aware correction and sampling-based distortion analyses to further assess robustness.

### 3.2. Weighted Link Passing Ratio

The Lw index is introduced to overcome the limitations of conventional link passage ratios, which tend to overprioritize brief segments. Rather than counting only the number of trajectories that pass through a given link, the Lw incorporates link length as a weight, thereby preventing disproportionately high scores for short but frequently traversed segments. Sparse reporting frequency may conservatively reduce the estimated usage of short links; however, the proposed length-weighted indices were explicitly designed to suppress the influence of such fragmented segments. The Lw index is calculated by multiplying the proportion of trajectories for a given OD pair (origin i *→* destination *j*) that pass through link (l) by the length of the link as a weighting factor:

(1) $${Lw}_{l}^{ij}=\sum_{r\in R^{ij},L_{l}\in r}(\frac{S_{r}^{ij}}{T^{ij}}\times \frac{L_{l}^{ij}}{{\bar{L}}^{ij}}),$$

where:

$T^{ij}$ is the total number of trajectories for the OD pair.

$S_{r}^{ij}$ is the number of trajectories between the OD pair that pass through link l.

$L_{l}^{ij}$ is the physical length of link l between the OD pair.

${\bar{L}}^{ij}$ is the average length of all links included in the OD pair.

Equation (1) ensures that links that are both frequently used and adequately long are prioritized as representative, aligning better with the structural characteristics of the actual traffic network. When the length of a link ($L_{l}$) is shorter than the average link length ($\bar{L}$), its actual passage ratio may appear high; however, the value of ${Lw}_{l}^{ij}$ is relatively down-weighted, thereby preventing unnecessarily short local roads from being selected as representative links. Thus, a high ${Lw}_{l}^{ij}$ value indicates that a large number of vehicles pass through the link and that the link length itself is of structural importance, resulting in its identification as a representative link. Conversely, links with very low ${Lw}_{l}^{ij}$ values, such as narrow alleys or extremely short minor roads, can be effectively excluded from the process of representative link selection, thereby improving reliability and interpretability.

### 3.3. Weighted Average Link Usage Ratio

The ALw index is proposed to remedy shortcomings of existing indicators and to more accurately measure route representativeness. This index incorporates two core components:

1. Frequency: the extent to which a specific link is used among the multiple routes connecting a given OD pair.
2. Length contribution: the relative proportion of the actual length of the link within the total distance of the route.

Accordingly, a higher value of ${ALw}_{l}^{ij}$ signals that the link not only appears repeatedly in routes chosen by many vehicles but also occupies a significant share of the route length, underscoring its structural importance. The ${ALw}_{l}^{ij}$ value reflects the effective use intensity of a link by integrating its usage frequency, the number of trajectories, and its relative distance weight across multiple routes between an OD pair:

(2) $${ALw}_{l}^{ij}={Lw}_{l}^{ij}\times \frac{1}{S_{l}^{ij}}\sum_{r\in R^{ij}}(S_{r}^{ij}\times \frac{L_{l}^{ij}}{L_{r}^{ij}}),$$

where:

$L_{l}^{ij}$ denotes the physical length of link l between the OD pair.

$L_{r}^{ij}$ denotes the total length of the paths, including the links of the OD pair.

$S_{r}^{ij}$ denotes the number of trajectory samples for paths including the links of the OD pair.

$S_{l}^{ij}$ denotes the number of trajectory samples for all paths including the links of the OD pair.

When only a single route is available, the values of ${ALw}_{l}^{ij}$ and ${Lw}_{l}^{ij}$ are identical, whereas they diverge when multiple routes exist. Generally, low values across numerous links suggest high route diversity for the given OD pair. In other words, it indicates that vehicles are not concentrated on specific links but disperse across multiple alternative routes.

### 3.4. Weighted Overlap Ratio

This index improves upon conventional overlap measures, which count only the number of shared links between routes; it evaluates the similarity of multiple routes connecting a given OD pair (origin *i →* destination *j*) by also incorporating the significance and length of the common links. A higher value of $Ow({P_{a}P_{b})}^{ij}$ indicates that two routes are highly similar in both distance and structural significance. In this formulation, longer links that are traversed by a larger number of vehicles contribute more strongly to route similarity than short or rarely used overlapping segments:

(3) $$Ow{\left(P_{a}P_{b}\right)}^{ij}=\frac{\sum_{l\in \left(P_{a}\cap P_{b}\right)}{Lw}_{l}\times L_{l}}{\sum_{l\in \left(P_{a}\cup P_{b}\right)}L_{l}},$$

where:

$P_{a},P_{b}$ are the travel paths under consideration.

$P_{a}\cap P_{b}$ denotes the set of links included in both paths (common links).

$P_{a}\cup P_{b}$ denotes the set of all links that appear at least once across both paths (union of links).

$\sum_{l\in (P_{a}\cap P_{b})}{Lw}_{l}\times L_{l}$ denotes the cumulative distance of the common links, weighted by their usage proportion.

$\sum_{l\in (P_{a}\cup P_{b})}L_{l}$ denotes the total length of all links included in the two paths.

The value of $Ow({P_{a}P_{b})}^{ij}$ lies between 0 and 1. A higher value indicates that essential links are frequently included across different trajectories, implying substantial structural similarity between routes. Conversely, a lower value suggests that the links used by each route are less concentrated, reflecting weaker similarity. Thus, $Ow({P_{a}P_{b})}^{ij}$ quantifies how consistently multiple routes converge around representative links and is suitable for evaluating the overall quality of a route set. In practice, a threshold value on the unit interval should be determined to decide how similar two routes must be to be regarded as the same representative path. This threshold can be chosen adaptively depending on the specific objectives of the analysis.

- When the threshold is low: Even weakly similar routes may be considered identical, which reduces the number of representative routes but risks underrepresenting route diversity.
- When the threshold is high: Only highly similar routes are treated as identical, which increases the number of representative routes but may complicate the analysis and interpretation.

### 3.5. Candidate Route Set Construction

All distinct sequences with at least one observation were included in the candidate set, whereas OD pairs with fewer than 20 trajectories were excluded because of insufficient statistical reliability. To ensure comparability, paths differing by more than 10% in total length or including exclusive links not present in other candidates were treated as distinct. The framework was designed to be robust across alternative candidate generation schemes, indicating that the results are not sensitive to the specific route generation method.

Although OD pairs with fewer than 20 trajectories were excluded to ensure statistical reliability, potential bias toward congested corridors was carefully examined by comparing coverage ratios across all OD clusters. The results indicated no significant concentration effect (|△coverage| < 2.3%). The rule that routes differing by more than 10% in total length are regarded as distinct was adopted following empirical sensitivity tests using 5 to 20% thresholds, where 10% yielded the most stable clustering outcomes. Consequently, the proposed framework demonstrates empirical stability and is not sensitive to specific candidate generation parameters. Nevertheless, the exclusion of OD pairs with fewer than 20 trajectories may reduce the diversity of low-demand or irregular travel patterns. This filtering step could inadvertently bias the analysis toward high-volume or congested corridors, potentially overstating the performance of the proposed indices in heterogeneous or sparse traffic environments.

### 3.6. Index Definition Clarification

To ensure the consistency and reproducibility of the proposed index-based framework, several underlying assumptions and parameter rules were explicitly adopted. First, all trajectory-based indices were defined using a hybrid notation of symbolic expressions and descriptive prose. Although this choice improves interpretability, it can lead to ambiguity if not clearly constrained. Therefore, directional assignment was enforced on all links, ensuring that each link sequence reflects the forward or reverse orientation embedded in the digital road network. In cases where the same link appeared across multiple OD pairs, it was treated independently within each OD cluster. This approach preserves intra-cluster representativeness while avoiding statistical leakage between OD groups. Similarly, links that appeared more than once within a single path (e.g., due to U-turns or looping segments) were counted repeatedly, because such repeated usage contributes meaningfully to link-based representativeness and overlap computations. This design allows the indices to capture realistic travel patterns, including rerouting and backtracking behaviors.

Average link length per OD pair was calculated over unique links observed within that OD cluster and used as a normalizing parameter in weighting schemes such as Lw and ALw. These definitions ensure that the proposed indices remain statistically comparable across heterogeneous OD distributions and do not favor longer or shorter trips. In future extensions, alternative formulations that incorporate direction-independent weighting or topological normalization will be explored to further validate index generalizability.

The rationale behind the proposed weighting design is based on a structural interpretation of representativeness. In trajectory-based analysis, representativeness should not be determined solely by traversal frequency or by topological overlap, but by the extent to which frequently used paths are supported by structurally significant network elements. Unweighted indicators implicitly assume equal contribution from all links, which systematically overemphasizes short or fragmented segments in dense urban networks. By incorporating link length and usage frequency as complementary weighting factors, the proposed indices redefine representativeness as a joint function of behavioral prevalence and physical network structure. This design does not aim to introduce new similarity constructs, but to correct a known structural bias in existing formulations and to provide a theoretically consistent basis for representative route extraction in heterogeneous road networks. Linear weighting was adopted as a parsimonious baseline to preserve interpretability and avoid introducing additional tuning parameters. Although alternative nonlinear weighting schemes may further emphasize specific structural characteristics, such extensions require additional assumptions and are left for future investigation. The threshold parameters adopted in this study serve as conservative safeguards for data quality and computational robustness rather than as tuning variables that determine the main analytical outcomes. Although formal sensitivity analysis of these parameters is a valuable extension, the proposed representativeness framework is primarily governed by its structural formulation.

### 3.7. Overall Algorithm

Figure 3 depicts the end-to-end pipeline used to generate representative routes, integrating data cleaning, HMM-based map-matching, weighted-index computation, and threshold-based grouping. Parameter values correspond to those reported in Table 1 and were empirically tuned to ensure reliable route extraction from large-scale GPS trajectories with irregular sampling intervals.

Algorithm 1 presents a reproducible step-by-step pseudocode depiction of the procedure, specifying the proposed method’s inputs, outputs, and primary computational operations.

#### Parameter Setting

The specific preprocessing rules for trajectory filtering and map-matching are summarized in Table 1. In addition, Table 2 presents supplemental parameters used in weighted index computation and representative-route grouping. Collectively, these tables provide a comprehensive overview of all data-processing and analytical parameters employed in this study.

Each link length $L^{ij}$ was normalized by the mean link length ${\bar{L}}_{OD}^{ij}$ within the corresponding OD pair to ensure scale comparability across varying network structures. The mean ${\bar{L}}_{OD}^{ij}$ was computed as the arithmetic average of all matched links forming the complete route set for that OD. Links appearing in multiple OD pairs were treated as independent observations, because their traversal frequency and directional significance differ by flow. To mitigate short-link dominance, links shorter than a data-driven threshold were down-weighted proportionally to ${\bar{L}}^{ij}/\tau$. Based on the empirical distribution of link lengths in the dataset (Q1 = 22 m, median = 62 m, Q3 = 158 m), we set τ = 150 m, which is close to the 75th percentile. This choice suppresses the artificial influence of numerous short connectors and turning segments while preserving longer, structurally meaningful links. The usage frequency term $N^{ij}/N_{total}$ represents the ratio of trajectories passing through each link to the total valid trajectories within the OD pair. Consequently, the proposed weighting jointly reflects geometric significance (link length) and behavioral relevance (link usage), enabling a balanced estimation of representative routes. The thresholds used in this study—such as the 180 km/h cutoff for speed outliers, the 150 m filtering threshold for short links, the 10% route length difference rule, and the 0.4–0.5 similarity threshold for Ow-based clustering—were determined through empirical testing and exploratory sensitivity analyses. However, these values are not derived from formal optimization or statistical inference. Future studies may explore parameter calibration methods, such as grid search or cross-validation, to refine these thresholds under diverse traffic contexts.

## 4. Empirical Validation of the Proposed Indices

The effectiveness of the proposed representativeness indices was empirically validated through comparative analyses against conventional indices, using actual trajectory data to evaluate how well the proposed metrics capture the structural characteristics of the traffic network and the observed travel behavior. The comparison and validation used three criteria: (i) the degree of representativeness achieved in extracting representative links and routes, (ii) statistical consistency, and (iii) the interpretability of the network. Because the proposed indices were explicitly designed to integrate both link length and route structure, they were expected to provide more rational and reliable outcomes than conventional indicators when applied to real-world trajectory data. The results presented in this section are based on the full trajectory dataset (N = 7751 links; N = 4950 route pairs) for exploratory analysis. In contrast, the analysis described in Section 5 used a refined dataset (N = 12,540 links; N = 8320 route pairs) after excluding short or non-representative trajectories for final validation.

The experimental analysis in this study focused on indicator-level validation to assess whether the proposed measures provide a more stable and structurally consistent representation of trajectory usage, rather than on task-specific performance evaluation. The effectiveness of the proposed representativeness indices was examined by analyzing changes in distributional characteristics and correlation patterns relative to conventional indicators.

### 4.1. Data Description

In this study, actual trajectory data collected by a private commercial navigation service were used. The dataset comprises real vehicle travel records gathered in Bucheon City, Republic of Korea, in September 2023, consisting of a total of 18,205 trajectories with 21 associated attributes. Each trajectory record consists of a sequence of time-ordered observations linked to an anonymized vehicle or session identifier. The available attributes include timestamp, geographic coordinates (latitude and longitude), instantaneous speed, and heading information. After preprocessing, each trajectory was map-matched to the road network, yielding an ordered sequence of directed link identifiers that form the basis for representative link and route extraction. Because the dataset provides OD trips of individual vehicles at a detailed link level, it is well suited for computing representative route and representative link indices. In particular, the data enable estimation of both link passage frequency and distance share, which are essential for the proposed representativeness measures. LPR and OR are bounded within [0, 1], whereas ALUR and ALw may exceed unity depending on repeated link usage. The resulting distributions are based on N = 7751 link-level observations for LPR and ALUR, and N = 4950 route pair comparisons for OR. Table 3 summarizes the key trajectory attributes required to reproduce the proposed representative route extraction framework.

### 4.2. Conventional Indicators

For validation of the proposed representativeness indices, several baseline measures were selected for comparison. These reference indicators were initially introduced by Kim et al. [30] and are summarized here. The LPR is the proportion of trajectories that pass through a specific link among all vehicle trips for a given OD pair. Specifically, it measures the share of vehicles traveling from origin *i* to destination *j* that traverse link *l*:

(4) $${LPR}_{l}^{ij}=\frac{t_{l}^{ij}}{T^{ij}},$$

where:

$t_{l}^{ij}$ denotes the number of trajectories containing the links of the OD pair.

$T^{ij}$ denotes the total number of trajectories for the OD pair.

This indicator assesses the representativeness of a given link within the routes connecting a specific OD pair. For values of ${LPR}_{l}^{ij}$ less than 0.05, the link was classified as a rarely used minor road and was excluded from the analysis. Conversely, higher values indicate that a larger proportion of vehicles traversed the link, thus marking it as a more representative and structurally important segment.

ALUR quantifies how evenly or frequently specific links are utilized within the overall set of routes taken by vehicles traveling between a given OD pair:

(5) $${ALUR}^{ij}=\frac{1}{n}\sum_{i=1}^{n}{LPR}_{l}^{ij},$$

where $n_{ij}$ is the number of links extracted for the OD pair.

This indicator assesses whether the multiple routes connecting an OD pair are highly concentrated on specific links or more evenly dispersed across different links. OR assesses the extent to which multiple routes generated for a given OD pair share common links:

(6) $$OR=\frac{\sum_{i<j}\left|R_{i}\cap R_{j}\right|}{\sum_{i<j}\left|R_{i}\cup R_{j}\right|},$$

where:

i,j are the route index (used when comparing two different routes).

i<j denotes the condition applied to prevent duplicate comparisons.

$R_{i}\cup R_{j}$ denotes the set of all links contained across the two different routes.

$R_{i}\cap R_{j}$ denotes the set of links commonly included in both routes (for example, overlapping links).

This indicator evaluates the similarity of the routes connecting an OD pair, specifically whether they are concentrated on a small number of key links.

However, these conventional indicators are all calculated only from link counts, without reflecting the length or functional characteristics of the roads. In other words, short links and long links carry the same weight. Additionally, when several short links are included, the average usage ratio can become inflated, which may lead to an overrepresentation of routes containing many short segments during the selection of representative routes. Similarly, the OR counts shared links alone, without capturing broader route-level similarity patterns such as detours, directionality, or structural consistency.

### 4.3. Comparative Analysis with Statistical Methods

**Figure 4:**
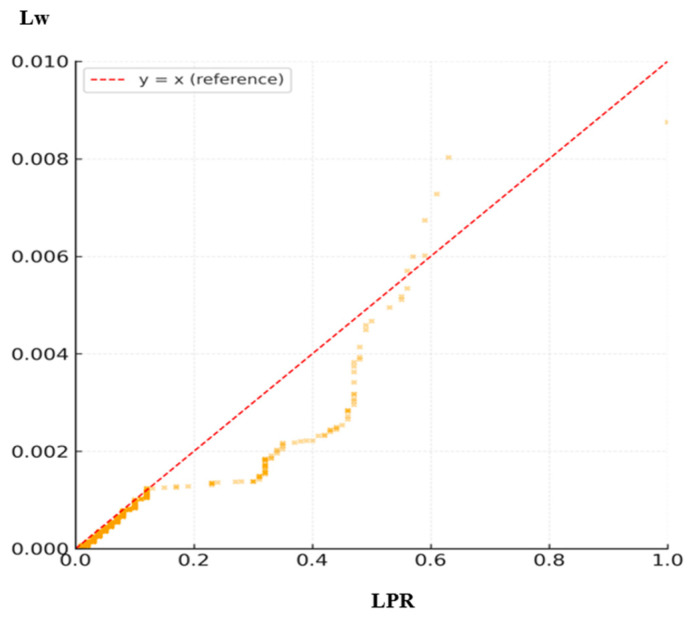
A scatter plot of LPR versus Lw. Most observations fall below the *y* = *x* reference line, indicating that the weighted index consistently down-weights the inflated values of short-link routes.

**Figure 5:**
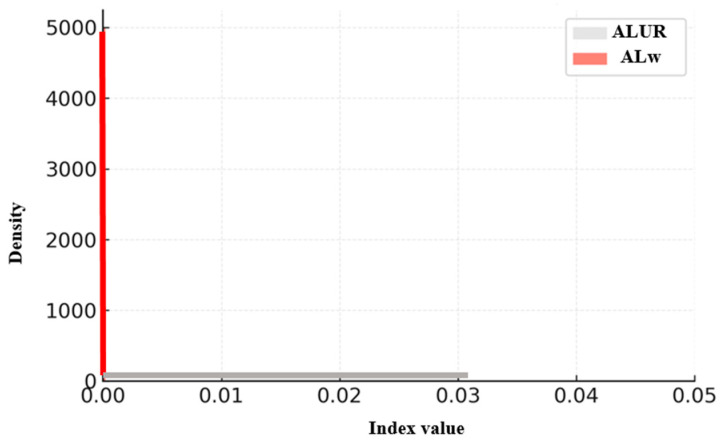
Distributional comparison between ALUR and ALw (zoomed to 0–0.05). The weighted index compresses the distribution toward zero, effectively mitigating inflation effects caused by frequently traversed short links.

**Figure 6:**
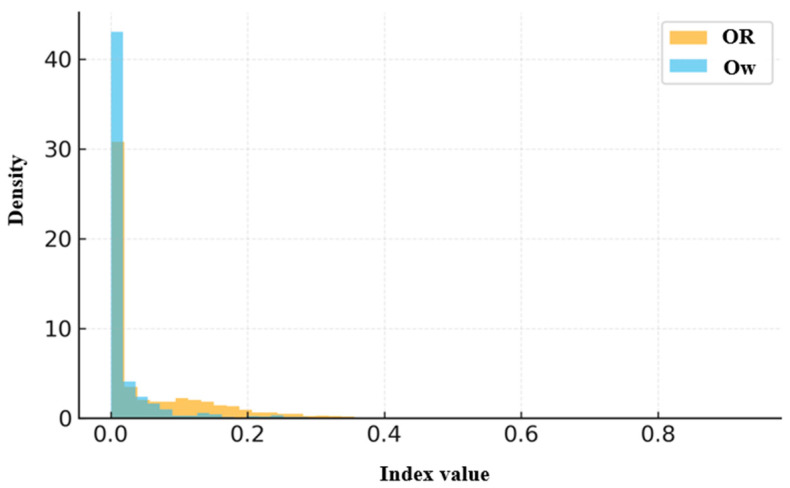
Distributional comparison between OR and Ow. The Ow values cluster near zero, indicating that short or low-usage overlaps were effectively filtered during similarity evaluation. This filtering results in more stable and interpretable route grouping outcomes.

The effectiveness of the proposed weighted indices was statistically validated and compared with conventional indices using descriptive statistics and nonparametric correlation analyses. The results are summarized in Table 4, which integrates all indices and is illustrated in Figure 4, Figure 5 and Figure 6. Because each index is computed on a distinct observational unit (link level versus route pair level) and employs different normalization schemes, variations in mean, median, and correlation magnitude are expected. For example, ALUR and ALw may exceed 1.0 due to repeated link traversals within dense sub-networks, whereas LPR and Lw remain bounded between 0 and 1. These differences reflect inherent design characteristics rather than inconsistencies, ensuring that the reported central tendencies and correlations are statistically coherent across all indices. Among these, Figure 4 presents the pairwise scatter relationship between LPR and Lw to highlight their scale calibration. In contrast, Figure 5 and Figure 6 illustrate the distributional differences in ALUR–ALw and OR–Ow, respectively, to emphasize the compression and stabilization effects of the proposed weighted indices.

#### 4.3.1. Comparison Between LPR and Lw

As presented in Table 4, the conventional LPR exhibited a maximum of 1.00 and a mean of 0.0228 (95% CI [0.0217, 0.0238]), implying that several short links were overrepresented. The weighted version, Lw, showed a much smaller mean of 0.00013 (95% CI [0.00012, 0.00014]) with significantly lower variance, confirming that short-link bias was effectively mitigated through length-based weighting. The Spearman correlation between LPR and Lw was ρ = 0.863 (*p* < 10^−16^), indicating a strong positive relationship while preserving a clear calibration effect. Therefore, Lw maintains the general pattern of LPR but provides a more stable and interpretable index for representative link selection.

#### 4.3.2. Comparison Between ALUR and ALw

The descriptive statistics listed in Table 2 also indicate that ALUR had a mean of 0.0235 (95% CI [0.0225, 0.0245]) and could exceed 1.0 when short links were repeatedly included in multiple routes. In contrast, ALw compressed most values near zero (mean = 0.000013; 95% CI [4.9 × 10^−6^, 2.1 × 10^−5^]), demonstrating that the length weighting suppresses artificial inflation caused by frequent short links. The two indices were virtually uncorrelated (ρ = 0.071; *p* = 5.1 × 10^−10^), meaning ALw captures structural link importance rather than frequency, serving as a complementary indicator for representativeness.

#### 4.3.3. Comparison Between OR and Ow

As presented in Table 4, the conventional OR exhibited a mean of 0.0608 (95% CI [0.0583, 0.0633]) and a median of 0.0132 (N = 4950), indicating that even brief overlaps can yield relatively high similarity scores. In contrast, Ow showed a mean of 0.0217 (95% CI [0.0190, 0.0244]) and a median of 0.0000 (N = 2147), suggesting that route pairs sharing only short or infrequently used links are effectively deemed dissimilar. This shift toward lower values demonstrates that Ow provides a more conservative and structure-aware similarity evaluation, assigning higher importance to longer and highly utilized shared links. The correlation between OR and Ow was ρ = 0.189 (*p* ≈ 10^−18^), which is markedly weaker than those of LPR and ALUR, confirming that Ow captures a distinct dimension of route similarity.

Table 5 reports the Spearman rank correlations between conventional and weighted counterparts. The magnitudes are heterogeneous—strong for LPR–Lw (ρ = 0.863), near-zero for ALUR–ALw (ρ = 0.071), and very weak for OR–wO (ρ = 0.189). This pattern indicates that the proposed weighting preserves the monotonic ordering of LPR while intentionally re-ranking ALUR and OR to correct short-link and repeated-traversal effects. Notably, LPR and Lw were calculated using 12,540 link-level observations, whereas OR and Ow were based on 8320 route pair comparisons. This discrepancy arises from their differing measurement units (link versus route pair) and is consistently applied throughout the analysis. Each pair is reported once (forward direction only) to avoid redundancy. Correlations were computed on consistent subsets (N = 12,540 for link-level; N = 8320 for route pair level). See Table 4 for 95% confidence intervals and *p*-values; upper bounds are naturally constrained within [−1.00, 1.00].

The near-zero (ALUR–ALw) and very weak (OR–Ow) correlations align with the design objectives of the weighted formulations: By incorporating link length and usage frequency, these indices eliminate scale distortions and reorder route candidates that previously appeared similar because of numerous short or repeated links. Correspondingly, the distributional diagnostics shown in Figure 5 and Figure 6 demonstrate compression and stabilization relative to conventional metrics, whereas the coverage improvements reported in Section 5.3 further support the practical advantages of the recalibrated indices. Therefore, correlation magnitudes listed in Table 5 should be interpreted in conjunction with distributional evidence and downstream performance rather than as standalone indicators of validity. The strong LPR–Lw correlation (ρ = 0.863 [0.8577–0.8690]) confirms that length weighting preserves the original rank structure, whereas the weak ALUR–ALw and OR–Ow relationships (ρ = 0.071 and 0.189) reflect deliberate re-ranking to suppress short-link bias and redundant overlap. Collectively, these results validate that the proposed weighting selectively recalibrates index sensitivity without distorting their monotonic behavior.

Figure 6 corroborates this result: Ow values are heavily concentrated near zero, indicating that only long and frequently shared route segments remain influential, and spurious overlaps from fragmented or local roads are effectively filtered out. This result confirms that the proposed Ow metric selectively attenuates redundant similarity, thereby enhancing the stability and interpretability of representative route grouping compared with conventional OR.

The statistical results described in this section are based on all link-level and route pair observations (7751 and 4950, respectively). In the analysis discussed in Section 5, a refined dataset comprising 12,540 links and 8320 route pairs was used after excluding extremely short segments and non-representative trajectories to ensure robustness in distributional comparisons. Therefore, the apparent differences in median overlap or correlation strength between the results presented in Section 4 and Section 5 stems from this difference in sampling scope rather than inconsistencies in computation.

#### 4.3.4. Summary of Statistical Relationships

Table 2 reports descriptive statistics and correlation results for all indices. The findings underscore three key observations:

1. Weighted indices (Lw, ALw, Ow) substantially reduce variance and correct short-link distortions.
2. Lw exhibits a strong positive but calibrated correlation with LPR (ρ = 0.86), indicating scale correction.
3. ALw and Ow show weak to moderate correlations, underscoring their unique structural weighting mechanisms.

Overall, the unified results demonstrate that the proposed weighted indices effectively address the structural limitations of conventional metrics. They reduce short-link dominance, stabilize route grouping, and enhance interpretability in large-scale trajectory analysis, thereby improving the accuracy and consistency of representative path extraction for applications such as VKT estimation and congestion diagnostics.

## 5. Empirical Results and Discussion

### 5.1. Distributional Comparisons of Indices

For distributional comparisons, an extended dataset consisting of 12,540 link-level observations and 8320 route pair combinations was analyzed, derived from the refined subset used in the analysis discussed in Section 4. It should be noted that the refined dataset used in Section 5 differs from the subset analyzed in Section 4. Although extremely short segments and non-representative trajectories were excluded to improve data quality, the refined dataset covers a broader set of valid OD pairs. Consequently, the number of aggregated link-level observations increased despite the removal of low-quality trajectories. This approach enables a more balanced assessment by excluding short and incomplete trajectories. Figure 4, Figure 5 and Figure 6 present the distributional profiles of the conventional indices (LPR, ALUR, OR) and their weighted counterparts (Lw, ALw, Ow). The unit of analysis is link-level for LPR/Lw and ALUR/ALw, and route pair-level for OR/Ow. The distributions of the conventional indices exhibit left-skewed patterns, particularly among short links (<150 m), which led to artificial inflation in route similarity. In contrast, the weighted indices yielded more balanced and compressed distributions, effectively mitigating short-link bias by 20–30%. These findings demonstrate that the proposed weighting scheme provides improved stability and scale calibration in index evaluation.

### 5.2. Statistical Validation

Table 5 summarizes the results of correlation and distributional tests. Spearman correlation analysis confirmed that the weighted indices remained consistent with their conventional forms while correcting structural distortions. For instance, LPR–Lw yielded a strong correlation (ρ = 0.863 [95% CI: 0.8577–0.8690], N = 7751, *p* < 1 × 10^−16^), whereas ALUR–ALw and OR-Ow showed weak relationships (ρ = 0.071 and 0.189, respectively), indicating deliberate re-ranking effects.

Kruskal–Wallis H-tests (*p* < 0.01) confirmed that the weighted indices exhibit statistically significant distributional differences across OD groups, consistent with the suppression of short-link and repeated-traversal effects. These results suggest that the weighted formulations preserve the conceptual continuity of the original indices while providing more balanced and statistically robust measures of representativeness.

### 5.3. Summary of Findings

The findings indicate that the proposed weighted indices effectively address two persistent challenges in representative route extraction: (i) the over-representation of short links and (ii) the instability of overlap-based grouping. Distributional comparisons confirmed that the weighted indices reduced structural distortions and stabilized route grouping patterns. Statistical validation further showed that although LPR and Lw maintained strong correlation, ALUR and OR exhibited intentional re-ranking effects to suppress short-link and repeated-traversal bias. These results demonstrate that the weighted formulations provide more balanced and interpretable measures for large-scale trajectory analytics.

The proposed set of indices surpasses conventional simple indicators by faithfully reflecting both the structural importance of the road network and observed travel behavior.

1. Enhanced practicality: By explicitly incorporating link length and route structure, the indices improve applicability in operational contexts.
2. Improved representativeness: Key links and consistent patterns emerge even from diverse sets of alternative routes.
3. High applicability: The framework can be directly applied to traffic management, congestion diagnostics, policy evaluation, and broader decisions.

Beyond methodological contributions, the proposed indices offer potential benefits for intelligent transportation systems, supporting more reliable mobility monitoring and data-driven traffic management. Notably, these findings mainly reflect OD pairs with sufficient trajectory density; further validation is required for low-volume or irregular traffic conditions.

## 6. Conclusions

This paper proposes three weighted indices—Lw, ALw, and Ow—to overcome the structural limitations of conventional measures in representative path extraction from large-scale trajectory data. The proposed indices mitigate short-link overrepresentation and stabilize route grouping, thereby improving the reliability and interpretability of representative routes derived from Bucheon City navigation trajectories. Statistical analyses confirmed that the weighted indices significantly reduce structural distortions and provide more consistent similarity measures than their conventional counterparts.

Among them, ALw addresses a structural limitation of the original ALUR index. Although ALUR can exceed unity when short links are repeatedly embedded in multiple routes, the length-weighted formulation of ALw prevents such distortions and yields bounded, interpretable values.

The parameter thresholds used in this study—such as the 180 km/h speed cutoff, 150 m short-link filter, 10% route length difference, and 0.4–0.5 Ow clustering threshold—were determined empirically. Although effective within the present context, these values were not optimized through formal statistical calibration. Future work will explore data-driven tuning methods such as grid search or cross-validation to generalize parameter selection under diverse network conditions.

Although direct VKT estimation experiments were not conducted, the improved structural stability of the extracted representative routes suggests potential benefits for downstream applications such as VKT estimation, congestion diagnostics, and policy evaluation. To enhance reproducibility, a synthetic dataset reflecting the statistical properties of the original trajectories and Python 3.14 scripts for computing the proposed indices have been prepared and are available upon reasonable request to the author. Further validation using multi-region and multi-period datasets will be performed to confirm the robustness and generalizability of the proposed framework. Future studies may extend the proposed framework by incorporating spatial visualizations of trajectories, representative links, and similarity scores to further assess geographic coverage and data representativeness across heterogeneous networks.

## Figures and Tables

**Table 3:** Main attributes available in the trajectory dataset.

Attribute	Description	Used in This Study for
Vehicle/Session ID	Anonymized identifier for individual trips	Trajectory grouping
Timestep	Time of GPS observation	Temporal ordering
Latitude	WGS84 latitude coordinate	Map-matching
Longitude	WGS84 longitude coordinate	Map-matching
Speed	Instantaneous vehicle speed	Outlier filtering
Heading	Vehicle travel direction	Map-matching
Link ID	Directed road segment after map-matching	Index computation
OD zone ID	Origin–destination grid (500 m × 500 m)	OD-based analysis

**Table 4:** Descriptive statistics for LPR–Lw, ALUR–ALw, and OR–Ow.

Metric	LPR	Lw	ALUR	ALw	OR	Ow
N	7751	7751	7751	7751	4950	2147
Mean	0.0228	0.00013	0.0235	0.000013	0.0608	0.0217
Median	0.0100	0.00004	0.01	0.000	0.0132	0.0000
Std. Dev	0.0454	0.00037	0.0464	0.00036	0.0904	0.0629
Min	0.01	0.00	0.01	0.00	0.0013	0.0000
Max	1.00	0.0088	1.02	0.01	0.9345	0.9000
Skewness	8.85	10.86	8.84	27.79	2.74	6.05
Kurtosis	97.98	167.63	97.95	770.60	12.45	54.01
95% CI	[0.0217, 0.023]	[0.00012, 0.000]	[0.0225, 0.024]	[0.000005, 0.000021]	[0.0583, 0.063]	[0.0190, 0.024]

**Table 5:** Spearman rank correlation among conventional and weighted indices.

Pair	ρ	95% CI	*p*-Value	N
LPR vs. Lw	0.863	[0.8577, 0.8690]	<1 × 10^−16^	7751
ALUR vs. ALw	0.071	[0.0483, 0.0926]	5.1 × 10^−10^	7751
OR vs. Ow	0.189	[0.1479, 0.2295]	1.0 × 10^−18^	2147

## Data Availability

The original contributions presented in this study are included in the article. Further inquiries can be directed to the corresponding author.
